# Caregiving Is Driving My Time: Caregiver and Care Recipient Driving Behaviors and Hours of Care

**DOI:** 10.1093/geroni/igab046.487

**Published:** 2021-12-17

**Authors:** Athena Koumoutzis, Jonathon Vivoda, Jiawei Cao

**Affiliations:** 1 Miami University, Oxford, Ohio, United States; 2 Miami University, Miami University, Ohio, United States

## Abstract

Informal caregivers often provide transportation assistance as older adult care recipients (CRs) begin regulating their driving (e.g., avoid certain driving situations, decrease/cease driving). This study examined how caregiver and CR driving frequency and CR’s driving avoidance behaviors impact caregiving intensity. Using data from Round 7 of the National Health and Aging Trends Study and the linked National Survey of Caregiving (n=1048 dyads), results indicated that caregiving intensity was highest among caregivers who drove everyday (5.38 hours) and for CRs who had not driven in the last month/did not drive (4.65 hours). Negative binomial regression techniques were used to assess and compare driving-related predictors. Compared to CRs who reported no avoidance of nighttime driving, caregivers of CRs who do not drive at all can expect to provide about 36% more hours of caregiving per day. Caregiving intensity was not significantly related to CR’s driving alone, on the highway, or in bad weather avoidance behaviors. CRs who drove every day, most days, and rarely required between 33% and 40% fewer expected hours per day of caregiving compared to CRs who had not driven in the past month. The expected number of hours spent providing care per day was 36% higher among caregivers who drove the care recipient every day, 28% higher among most-day drivers, and 30% higher among those who never drove as opposed to caregivers who drove some days per week. Results suggest that caregiving intensity is related more to caregiver and CR driving frequency than CR driving avoidance behaviors.

